# Effects of Chemotherapy on Aneuploidy Rates in Sperm from Male Patients with Testicular Cancer or Hodgkin’s Lymphoma—A Systematic Review

**DOI:** 10.3390/jcm13133650

**Published:** 2024-06-22

**Authors:** Jasmin Nissi, Laila Kalam, Laura Catalini, Jens Fedder

**Affiliations:** 1 Centre of Andrology and Fertility Clinic, Odense University Hospital, DK-5000 Odense, Denmark; janis22@student.sdu.dk (J.N.); lakal22@student.sdu.dk (L.K.); laura.catalini@studenti.unicam.it (L.C.); 2 Department of Clinical Research, Faculty of Health Sciences & Medicine, University of Southern Denmark, DK-5000 Odense, Denmark

**Keywords:** cancer, chemotherapy, aneuploidy, male, spermatogenesis

## Abstract

Background: Testicular cancer and Hodgkin’s lymphoma are prevalent malignancies among young males aged 20 to 39. The incidence of testicular cancer and lymphoma has risen in recent years, with orchiectomy often followed by adjuvant chemotherapy as the primary treatment for testicular cancer and chemotherapy for lymphoma. Chemotherapy has been associated with an increased risk of aneuploidy and reduced fertility. Method: This systematic review included seven studies, both case–control and longitudinal prospective designs, from the PubMed, Embase, and Cochrane Library databases. The screening process was conducted using the online tool covidence.org. Results: The study outcomes indicate varied impacts of chemotherapy on aneuploidy rates. An increase in the aneuploidy rates, notably for the sex chromosomes, immediately post-treatment was a common trend, followed by a decline in pretreatment values. Conclusion: This systematic review presents the effects of chemotherapy on the aneuploidy rates of testicular cancer and Hodgkin’s lymphoma patients, with a decrease post-treatment. The findings underscore the need for larger, well-designed studies with a longer study period.

## 1. Introduction

Testicular cancer and lymphoma are the most common types of malignant tumors among young males between the ages of 20 and 39 years. Testicular cancer is classified into two types based on the cancerous cells: germ cell tumors (GCTs) and non-germ cell tumors (gonadal or sex-cord stromal tumors). Germ cell tumors, which are further divided into seminomatous and non-seminomatous, have been the most reported form of malignant tumors arising in the testes [1]. Lymphoma disease is divided into two main types: Hodgkin’s and non-Hodgkin’s lymphoma. Hodgkin’s lymphoma is the most common form among young men [2]. The incidence of testicular cancer has been increasing in recent years, with a low mortality rate [3]. The first-line treatment for testicular cancer is often orchiectomy, with additional postsurgical treatment and adjuvant chemotherapy to prevent relapse of the disease [4]. The most common treatment form for lymphoma is chemotherapy, with a high survival rate. Although chemotherapy is an effective treatment option for lymphoma, it can have severe adverse effects on the patients due to the presence of alkylating agents. Alkylating agents can be associated with an increased risk of infertility due to decreases in the sperm concentrations of young boys and adolescents treated for cancer [5].

The human genome normally consists of 46 chromosomes arranged in 23 pairs, with 1 chromosome in each pair inherited from each parent. Aneuploidy is defined as an abnormality in the number of chromosomes in a cell, encompassing conditions characterized by trisomy (an additional chromosome) or monosomy (a missing chromosome). Notable instances of trisomy include Down syndrome (resulting from an additional chromosome 21), Edwards syndrome (associated with an extra chromosome 18), Patau’s syndrome (stemming from an additional chromosome 13), Klinefelter syndrome (an additional X chromosome in males) [6], and Triple X syndrome (trisomy X). Conversely, an example of monosomy is evident in Turner syndrome (monosomy X) [7].

While chemotherapy can play an important role in the treatment of testicular cancer and lymphoma, it can also have unintended long- and short-term side effects on the body of the patient due to the toxicity components of the treatment, including gonadal toxicity [8].

Chemotherapy is composed of two to four cycles of the PEB (cisplatin, etoposide, and bleomycin) regimen. Some studies have documented the effects of PEB chemotherapy on the inhibition of spermatogenesis. However, only a few studies have documented the effects of chemotherapy on sperm aneuploidy rates.

The aim of this systematic review is to describe the effects of chemotherapy on aneuploidy rates.

## 2. Materials and Methods

### 2.1. Systematic Review

#### 2.1.1. Eligibility Criteria

Our focus was on original papers and studies that included male sex populations and their aneuploidy rates before and after chemotherapy. Eligible studies were limited to the Scandinavian language or English language. Review articles were excluded. Studies focusing on the female population and animal studies were excluded due to having the wrong study population. Studies not reporting aneuploidy rates were excluded due to focus on the wrong outcome. No constraints were imposed on the study year, country, or publishing journal.

#### 2.1.2. Information Sources and Search String

Three different electronic databases were searched. The electronic database Pubmed.org was our primary search database, in which an advanced search was conducted. The final advanced search was conducted in October 2023 with the following search string: ((Chemotherapy OR Radiotherapy OR Anticancer therapy OR Antineoplastic therapy) AND (Aneuploidy OR Aneuploidy syndrome OR Genetic OR DNA OR DNA fragmentation OR DNA damage OR Chromosomal abnormality OR Chromosomal anomaly OR Chromosomal aberration) AND (Spermatogenesis OR Sperm fluid OR Semen quality OR Seminal fluid OR Sperm chromosome OR Semen fraction OR Spermatozoa) AND (Fertility OR fertility male OR Infertility male OR Childhood puberty OR Adolescent OR Haploidy)).

The same keywords and terms were used to conduct other searches in Embase and Cochrane Library.

#### 2.1.3. Study Selection and Data Collection Process

The screening process was conducted using the online tool covidence.org. The initial results were screened according to the titles and abstracts by two investigators. Subsequently, the full texts of the articles were screened, and studies not fulfilling the inclusion criteria were excluded, leaving the remaining studies for data extraction.

The data of the eligible studies were extracted and evaluated by two investigators. The outcome of the research question was the effect on aneuploidy rates before and after chemotherapy.

#### 2.1.4. Risk of Bias in Individual Studies

Randomization of the study populations in the case–control and longitudinal articles included in this review was difficult to obtain due to the research question. Therefore, a risk of bias was carried out for the individual studies. The longitudinal prospective studies were evaluated using the risk-of-bias tool by the CLARITY group at McMaster University [9]. For the case–control studies, the evidence was evaluated using the CASP checklist [10].

## 3. Results

### 3.1. Study Selection

**Figure 1:**
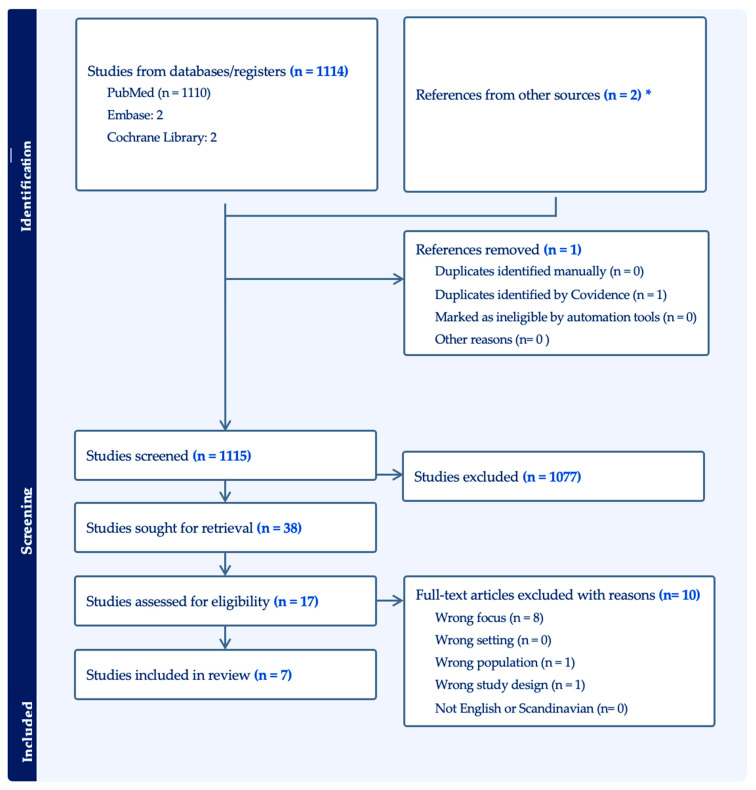
PRISMA flowchart diagram. * The initial search in the databases yielded a total of 1114 studies, with an additional 2 papers obtained through expert knowledge.

The study selection process is presented in the PRISMA flowchart diagram (Figure 1). We obtained 1114 articles after the initial search string on PubMed, Cochrane Library, and Embase, with an additional 2 papers obtained through expert knowledge. Only 1 duplicated article was removed, 1115 articles were screened by title and abstract, and we excluded 1077 articles. This resulted in 38 articles that underwent full screening and 10 articles that were not included in the screening due to missing eligibility criteria. The total number of articles that were included in the following review is seven.

### 3.2. Study Characteristics

An overview of the study characteristics is provided in Table 1. The longitudinal prospective studies included one Italian and two French studies [11,12,13]. The case–control studies included one French, one French/Lebanese, one American, and one Canadian study [8,14,15,16]. The primary outcomes of all the studies included in this review were the effects of chemotherapy on aneuploidy rates. The study period was from 1997 to 2017.

### 3.3. Study Outcomes

In a longitudinal prospective study performed by Burrello et al. [12], they investigated the effects of antineoplastic treatment on the sperm aneuploidy rates and sperm concentrations in male offspring diagnosed with testicular cancer at 3, 6, 9, 12, 18, 24, and 36 months from the treatment time. Analysis of the sperm aneuploidy rates was conducted using a double and triple multicolor fluorescence in situ hybridization (FISH) technique targeting chromosomes 8, 12, 18, X, and Y.

A total of 11 males between 18 and 30 years of age with testicular cancer and 18 healthy males between 19 and 35 years of age participated. The results showed that there was a slight increase in the aneuploidy rates at 6 months after treatment, but 36 months after treatment, the results showed a significant decrease in the total aneuploidy rates (%) ((0.65) *p* = 0.005) compared to the pretreatment value ((1.01) *p* = 0.005).

Another French longitudinal prospective study from 2001 [11] investigated the effects of PEB adjuvant chemotherapy on the aneuploidy rates 6 to 18 months after performing the treatment in testicular cancer patients.

The FISH technique was used for chromosomes 7, 16, 18, X, and Y. The results of the study showed an increase in diploidy and disomy for chromosomes 16, 18, and XY after chemotherapy, in comparison to the control group (Table 2 and Table 3).

In a case–control study performed in Canada [14], human sperm chromosome complements were observed in four testicular cancer patients before and 2 to 13 years after receiving bleomycin, etoposide, and cisplatin (BEP) chemotherapy treatment. The main outcome was no notable disparity in the total frequency of sperm chromosomal abnormalities, with 10.2% before chemotherapy and 10.7% after chemotherapy. The results for the frequency of numerical abnormalities were similar, with 2.5% before chemotherapy and 2.4% after chemotherapy.

In a French case–control study [16], the frequency of sperm aneuploidy in Hodgkin’s disease patients was investigated using fluorescence in situ hybridization (FISH). Chromosomes 1, 6, 11, X, and Y were studied, and results were obtained both before and after administering chemotherapy. Overall, all chromosomes had an increase in the aneuploidy rate both before and after chemotherapy. At day 0, the frequencies of 24 and XX and 24 and XY (1.64% and 8.46%, respectively) were higher than in the control group. Hyperhaploid 24, YY, and disomy 1 also presented higher values. The post-treatment aneuploidy rate after 38 days showed an increase in all chromosomes studied, with 24 and XX and 24 and XY standing out with rates more than three times higher compared to the control group.

In a French prospective longitudinal study, Martinez et al. [13] investigated the effects of doxorubicin, bleomycin, vinblastine, and dacarbazine (ADVP) chemotherapy (±radiotherapy) and doxorubicin, cyclophosphamide, vincristine, and prednisone (CHOP)/mechlorethamine, oncovin, procarbazine, prednisone–doxorubicin, bleomycin, and vinblastine (MOPP-ABV) (±radiotherapy) on aneuploidy rates and sperm production in Hodgkin’s and non-Hodgkin’s lymphoma patients. The FISH method was used before and 3, 6, 12, and 24 months after administering antineoplastic treatment.

Before treatment (month 0), the aneuploidy rates were higher in Hodgkin’s lymphoma patients in comparison to the control group, especially hyperhaploid XY (*p* = 0.008) and disomy 18 (0.10). Furthermore, the aneuploidy rates continued to increase after 3 months, significantly for haploid 24 and XY. Lower levels were found after 12 and 24 months post-treatment.

In an American case–control study [15], differences in the aneuploidy rates before and after chemotherapy in patients with testicular cancer or Hodgkin’s lymphoma were observed.

Increases in the aneuploidy rates for chromosomes 13 and 21 were observed for testicular cancer patients, with a significant increase in chromosome 21 (0.19%) before chemotherapy in comparison to its value at 12 (0.09%) and 18–24 months (0.08%). Also, increases in disomy 13 were observed at 18–24 months post-CT (0.22) compared to pre-CT and at 6 months post-CT (0.11 and 0.06).

With Hodgkin’s lymphoma, nullisomy 13 increased in 6 months (0.37) and 12 months (0.30). A decline to 0.27 was found after 18–24 months, reaching a level close to the pretreatment value (0.24). For nullisomy 21, an increase in aneuploidy was also observed, with a pretreatment value of 0.17% compared to the post-treatment value at 6 months (0.25%). A declined aneuploidy rate was observed after 12 months (0.15%).

For the sex chromosomes, the frequency of XY disomy was higher 6 months after treatment for both testicular cancer and Hodgkin’s lymphoma patients (0.21% and 0.28%, respectively) compared to the healthy male control group (0.13).

In the French–Lebanese case–control study [8], the aneuploidy rates were analyzed using FISH after neoplastic treatment in testicular cancer patients and lymphoma patients. No increases in the aneuploidy rates of 12 lymphoma patients were obtained except in one patient for disomies 13, 13, and 21 (0.79%), disomies X, Y, and 18 (1.45%) and in diploidy (1.38). Aside from four testicular cancer patients, no overall significant increase in the disomies of chromosomes 13, 21, 18, X, Y, or diploidy was observed.

### 3.4. Level of Study Evidence

Evaluation of the study evidence was carried out using the risk-of-bias tool by the CLARITY group at McMaster University for the retrospective longitudinal studies and the CASP checklist for the case–control studies (Table 4). All studies except one [15] had small sample sizes, thus not representing the general population.

Due to the small, simple sizes, there is an increased possibility of selection bias. The lack of consistent reporting standards across studies also hinders a comprehensive risk-of-bias assessment. Some studies also lacked clarity in their methodological descriptions, particularly regarding how they controlled for confounding variables. In conclusion, the tools to assess the risk of bias highlighted the potential for information bias and issues with the measurement of outcomes.

## 4. Discussion

This systematic review included seven case–control and longitudinal prospective studies, with an aim to describe the effects of chemotherapy on aneuploidy rates before and after treatment in patients diagnosed with testicular cancer and Hodgkin’s lymphoma. The quality of the study evidence among the included studies was low, primarily due to their limited sample populations.

Among the studies focusing on testicular cancer patients, some reported negligible or small correlations between chemotherapy and increased aneuploidy rates [8,13]. In contrast, other studies demonstrated a significant association between chemotherapy and increased aneuploidy rates [11,12,15]. Notably, Burrello et al. [12] observed higher pretreatment aneuploidy rates compared to the controls. The observed trend across the studies indicates a transient increase in aneuploidy rates shortly after chemotherapy, followed by a subsequent decline toward pretreatment values (Table 4). All chromosomes analyzed exhibited susceptibility to chemotherapy-induced alterations, with sex chromosomes, particularly disomy XY, appearing to be the most affected, as described by Tempest et al. [15], Burrello et al. [12], and De mas et al. [11].

Four articles studied the effects of chemotherapy on Hodgkin’s lymphoma patients [8,13,15,16]. One of the four studies, a case–control study [16], showed a correlation between chemotherapy and increased aneuploidy rates in the numerical chromosomes and sex chromosomes. These results were corroborated by Tempest et al. [15], who reported an elevated frequency of aneuploidy for autosomal chromosomes (13, 21) and sex chromosomes (disomy XY) in sperm. Additionally, both Tempest et al. [15] and Martinez et al. [13] reported higher pretreatment values for aneuploidy frequency. However, Thomas et al. [8] did not observe increased aneuploidy rates among Hodgkin’s lymphoma patients, with only 1 out of 14 patients exhibiting elevated rates.

Out of the seven studies included in this review, four longitudinal prospective studies were strengthened. One of them included a large prospective study of high quality [13]. Additionally, one of the case–control studies used a blind analysis to obtain their results [14]. The other studies could have had an increased risk of selection bias due to the limitations of their study populations. The lack of consistent reporting standards across the studies also hindered a comprehensive risk-of-bias assessment.

Some strengths of this study include the careful search in three databases and the process following the recommendations set by the PRISMA guideline. To our knowledge, this review, therefore, contains the highest number of studies in this field to date. This study included patients who received all kinds of chemotherapy and had different aneuploidies, including all chromosomes.

A limitation of our review is that many of the published studies are small and of poor quality, leading to low statistical power and an increased risk of type II errors. Not all studies included semen samples obtained before treatment, and therefore, it was difficult to distinguish between the effects of the disease and the effects of the antineoplastic treatment. Furthermore, it could also be considered if other confounders should have been included in the studies.

Increased evidence about sperm aneuploidy after chemotherapy may advance the field of andrology. When sufficient knowledge is obtained, the counseling of patients according to their sperm aneuploidy rates is highly relevant before administering chemotherapy.

Future studies should aim for larger sample populations to improve the reliability and applicability of the results. Standardized reporting guidelines should be adopted in future research to enhance transparency and reproducibility with larger samples and by ensuring comprehensive documentation of their methods, including detailed descriptions of how confounders are managed.

## 5. Conclusions

In conclusion, most of the studies showed correlations between antineoplastic treatment and increased aneuploidy rates. Importantly, in some studies, an increased aneuploidy frequency was observed prior to antineoplastic treatment for both testicular cancer patients and Hodgkin’s lymphoma patients. These patients also showed increases in their aneuploidy rates shortly after treatment.

The evidence suggests that chemotherapy is an important factor that influences the rise in aneuploidy rates more than cancer itself, with a notable increase immediately after the end of treatment, followed by a decline toward pretreatment values within 38 days to over 1 year. While all chromosomes were affected in different proportions, the sex chromosomes demonstrated heightened susceptibility.

While each study in this review provides valuable insights, it is important for larger, well-designed studies with extended follow-up to inform clinical practice and guide interventions to prevent male infertility in patients undergoing antineoplastic treatment for both testicular cancer and Hodgkin’s lymphoma. A comparison of different alkylating and non-alkylating agents is absolutely needed.

## Figures and Tables

**Table 1:** Overview of study characteristics.

Author/Study Year	Country	Study Design	Cancer Type	Primary Outcome of Study
Martin. 1998 [14]	Canada	Case–control study	Testicular cancer	Effects of chemotherapy on sperm chromosome and infertility. Chromosomes: 1, 2, 4, 9, 12, 15, 16, 18, 20, 21, X, and Y.
De Mas et al., 2001 [11]	France	Longitudinal prospective study	Testicular cancer	Increased aneuploidy in testicular cancer after PEB treatment. Chromosomes: 7, 16, 18, X, and Y.
Thomas et al., 2004 [8]	France and Lebanon	Case–control study	Testicular cancer and lymphoma	Sperm aneuploidy rate after anticancer therapy. Chromosomes: X, Y, 13, 18, and 21.
Tempest et al. [15]	USA	Case–control study	Testicular cancer and lymphoma	Sperm aneuploidy frequency analyzed before and after chemotherapy.
Burrello et al., 2011 [12]	Italy	Longitudinal prospective study	Testicular cancer	Effects of antineoplastic treatment on aneuploidy rate in testicular cancer. Chromosomes 8, 12, 18, X, and Y.
Monteil et al., 1997 [16]	France	Case–control study	Hodgkin’s Lymphoma	Increased aneuploidy frequency in spermatozoa from Hodgkin’s disease patients after chemotherapy. Chromosomes affected: 1, X, Y, 6, and 11.
Martinez et al., 2017 [13]	France	Longitudinal prospective study	Lymphoma	Effect of chemotherapy on aneuploidy rate in male patients diagnosed with lymphoma before and after treatment.

**Table 2:** Aneuploidy rates (in %) before chemotherapy treatment in testicular cancer and Hodgkin’s lymphoma patients.

Author/Study	Cancer Type	N (Men)		Aneuploidy Rate before Treatment (in %) (Testicular Cancer)		Aneuploidy Rate before Treatment (in %) (Hodgkin’s Lymphoma)	
Martin. 1998 [14]	Testicular cancer	Control 12	Case 4	Sperm chromosomal abnormalities: 10.2 ** numerical abnormalities: 2.5 **			
De mas et al., 2001 [11]	Testicular cancer	**Control** 5	**Case** 5	**Control** NR *	**Case** NR *		
Thomas et al., 2004 [8]	Testicular cancer and lymphoma	**Control** 12 **Case** 12 (Testicular cancer) 12 (Lymphoma)		**Control** 13, 13, 21: 0.40 12, 21, 21: 0.30 X, X, 18: 0.16 Y, Y, 18: 0.18 X, Y, 18: 0.45 X, Y: 0.15 Diploidy: 0.54	**Case** NR *	**Control** 13, 13, 21: 0.40 12, 21, 21: 0.30 X, X, 18: 0.16 Y, Y, 18: 0.18 X, Y, 18: 0.4 X, Y: 0.15 Diploidy: 0.54	**Case** NR *
Tempest et al., 2008 [15]	Testicular cancer and lymphoma	**Control** 10 **Case** 5 (Testicular cancer) 5 (Lymphoma)		**Control** X: 49.08 Y: 50.13 Disomy XX: 0.02 Disomy XY: 0.18 Disomy YY: 0.01 Diploid: 0.15 Null: 0.58 13: 0.16 21: 0.15	**Case** X: 48.12 Y: 50.94 Disomy XX: 0.02 Disomy XY: 0.25 Disomy YY: 0.03 Diploid: 0.17 Null: 0.64 13: 0.11 21: 0.19	**Control** X: 48.18 Y: 50.5 Disomy XX: 0.05 Disomy XY: 0.19 Disomy YY: 0.02 Diploid: 0.25 Null: 1.06	**Case** X: 49.08 Y: 50.13 Disomy XX: 0.02 Disomy XY: 0.18 Disomy YY: 0.01 Diploid: 0.15 Null: 0.58
Burrello et al., 2011 [12]	Testicular cancer	**Control** 18	**Case** 11	**Control** 1.38	**Case** 1.64		
Monteil et al., 1997 [16]	Hodgkin’s lymphoma	**Control** 2	**Case** 1			**Control** 23,Y: 49.30 23,X: 49.30 24,XX: 0.08 24,YY: 0.05 24,XY: 0.80 24,X or Y + 1: 0.27 46,XY: 0.08 46,XX: 0.07 46,YY: 0.03 Normal/6/11: 99.23 Disomy 6: 0.08 Disomy 11: 0.22 Diploid: 0.27	**Case** 23,Y: 46.41 23,X: 41.84 24,XX: 1.64 24,YY: 0.22 24,XY: 8.46 24, X or Y + 1: 0.56 46,XY: 0.62 46XX: 0.07 46,YY: 0.14 Normal/6/11: 97.7 Disomy 6: 0.28 Disomy 11: 0.80 Diploid: 1.0
Martinez et al., 2017 [13]	Lymphoma	**Control** 29	**Case** 74			**Control** Haploid: 99.45 24,XY: 0.26 24,YY: 0.06 24,XX: 0.0424, X/Y+18: 0.08 Disomy: 0.48 Diploidy *: 0.08	**Case** Haploid: 99.23 24 XY: 0.36 24,YY: 0.06 24,XX: 0.0624, X/Y+18: 0.12 Disomy: 0.66 Diploidy: 0.10

* NR: Not reported. ** The article provided the result in textual format and not the precise chromosomes affected.

**Table 3:** Aneuploidy rates (in %) after chemotherapy treatment in testicular cancer and Hodgkin’s lymphoma patients.

Author/Study	Cancer Type	N (Men)		Aneuploidy Rate after Treatment (in %) (Testicular Cancer)		Aneuploidy Rate after Treatment (in %) (Hodgkin’s Lymphoma)	
Martin [14]	Testicular cancer	**Control** 12	**Case** 4	Sperm chromosomal abnormalities: 10, 2 **** numerical abnormalities: 2, 4 ****			
De mas et al. [11]	Testicular cancer	**Control** 5	**Case** 5	**Case** Disomy 7: 0.07 Disomy 16: 0.09 Disomy 18: 0.044 ** Disomy X: 0.028	Disomy Y: 0.032 Disomy XY: 0.186 *** Diploidy (a): 0.254 *** Diploidy (b): 0.274 ***		
Thomas et al. [8]	Testicular cancer and lymphoma	**Control** 12 **Case** 12 (Testicular cancer) 12 (Lymphoma)		**Case** 13, 13, 21: 0.63 12, 21, 21: 0.56 X, X, 18: 0.29 Y, Y, 18: 0.49 X, Y, 18: 1.01	X or Y: 0.44 Diploidy: 1.14	**Case** **5 months–7 years** 13, 13, 21: 0.63 12, 21, 21: 0.56 X, X, 18: 0.29 Y, Y, 18: 0.49	X, Y, 18: 1.01 X or Y: 0.44 Diploidy: 1.14
Tempest et al. [15]	Testicular cancer and lymphoma	**Control** 10 **Case** 5 (Testicular cancer) 5 (Lymphoma)		**Case** **6 months** X: 48.81 Y: 50.43 Disomy XX: 0.02 Disomy XY: 0.2 Disomy YY: 0.01 Diploid: 0.15 Null: 0.51 13: 0.06 21: 0.28 **12 months** X: 48.24 Y: 50.98 Disomy XX: 0.01 Disomy XY: 0.15 Disomy YY: 0.00	Diploid: 0.21 Null: 0.63 13: 0.07 21: 0.17 **18–24 months** X: 48.73 Y: 50.35 Disomy XX: 0.02 Disomy XY: 0.23 Disomy YY: 0.04 Diploid: 0.17 Null: 0.65 13: 0.22 21: 0.08	**Case** **6 months** X: 48.71 Y: 50.23 Disomy XX: 0.02 Disomy XY: 0.28 Disomy YY: 0.02 Diploid: 0.24 Null: 0.75 **12 months** X: 47.89 Y: 51.14 Disomy XX: 0.03 Disomy XY: 0.19 Disomy YY: 0.02 Diploid: 0.35 Null: 0.73	**18–24 months** X: 49.13 Y: 49.86 Disomy XX: 0.02 Disomy XY: 0.31 Disomy YY: 0.02 Diploid: 0.43 Null: 0.67
Burrello et al. [12]	Testicular cancer	**Control** 18	**Case** 11	Chromosome 18, 12, X, and Y 3 months: 1.14 (SD 0.014) 6 months: 1.9 (SD 0.421) 9 months: 2.24 (SD 1.342) 12 months: 1.96 (SD 0.77) 18 months: 0.725 (SD 0.12) 24 months: 0.54 (SD 0.085) 36 months: 0.55 (SD 0.1)			
Monteil et al. [16]	Hodgkin’s lymphoma	**Control** **2**	**Case** **1**			**Case** **38 months** 23Y: 54.00 23X: 38.40 24XX: 1.06 24YY: 0.24 24XY: 4.60 24, X or Y +1: 0.80 46 XY: 0.38 46XX: 0.23 46YY:0.20 Normal/6/1: 98.08	DIsmy 6: 0.15 Disomy 11: 0.80 Diploid: 0.8246 XY: 0.62 46XX: 0.07 46YY:0.14 Normal/6/11: 97.7 Dismy 6: 0.28 Disomy 11: 0.80 Diploid: 1.0
Martinez et al. [13]	Lymphoma	**Control** **29**	**Case** **74**			Case *a: **3 months** Haploid: 99.16 24, XY: 0.51 24, YY: 0.04 24, XX: 0.0424, X/Y + 18: 0.09 Disomy: 0.70 Diploidy: 0.08 **6 months** Haploid: 99.42 24, XY: 0.33 24, YY: 0.02 24, XX: 0.03 24, X/Y + 18: 0.08 Disomy: 0.52 Diploidy: 0.08 **12 months** Haploid: 99.52 24, XY: 0.27 24, YY: 0.04 24, XX: 0.04 24, X/Y + 18: 0.06 Disomy: 0.40 Diploidy: 0.10 **24 months** Haploid: 99.59 24, XY: 0.23 24, YY: 0.04 24, XX: 0.04 24, X/Y + 18: 0.06 Disomy: 0.32 Diploidy: 0.06	Case *b: **3 months** Haploid: 97.80 24, XY: 1,41 24, YY: 0.11 24, XX: 0.08 24, X/Y + 18: 0.23 Disomy: 1.84 Diploidy: 0.35 **6 months** Haploid: 95.36 24, XY: 3.16 24, YY: 0.18 24, XX: 0.18 24, X/Y+18: 0.40 Disomy: 0.69 Diploidy: 03.94 **12 months** Haploid: 99.17 24, XY: 0.51 24, YY: 0.06 24, XX: 0.05 24, X/Y + 18: 0.11 Disomy: 0.72 Diploidy: 0.13 **24 months** Haploid: 99.37 24, XY: 0.38 24, YY: 0.04 24, XX: 0.04 24, X/Y + 18: 0.05 Disomy: 0.52 Diploidy: 0.08

* Own calculation with standard deviation (SD). ** *p* < 0.01, *** *p* < 0.001 a: chromosome 7 and 16 b: Chromosomes X, Y, and 18. **** The article provided the result in textual format, and not the precise chromosomes affected.

**Table 4:** Evaluation of study evidence using the risk-of-bias tool by the CLARITY group at McMaster University for the retrospective longitudinal studies and the CASP checklist for the case–control studies.

**Tool to Assess Risk of Bias by Clarity Group at McMaster University for Retrospective Longitudinal Study**												
**Study**	**Did the Study Address a Clearly Focused Issue?**	**Did the Authors Use an Appropriate Method to Answer Their Question?**	**Were the Cases Recruited in an Acceptable Way?**	**Were the Controls Selected in an Acceptable Way?**	**Was the Exposure Accurately Measured to Minimize Bias?**	**(a) Aside from the Experimental Intervention, were the Groups Treated Equally?**	**(b) Have the Authors Taken Account of the Potential Confounding Factors in the Design and/or in Their Analysis?**	**How Large was the Treatment Effect?**	**How Precise was the Estimate of the Treatment Effect?**	**Do you Believe the Results?**	**Can the Results be Applied to the Local Population?**	**Do the Results of this Study Fit with Other AvailableEvidence?**
Tempest et al., 2008 [15]	+	+	−	−	−	+	+			+	−	+
Martin. 1998 [14]	+	+	−	−	+	+	+				−	−
Monteil et al., 1997 [16]	+	+	−	−	−	+	+			+	−	+
Thomas et al., 2004 [8]	+	+	−	−	−	+	+				−	−
**CASP Checklist for Case–Control Studies**												
**Study**			**Is the Source Population (Sampling Frame) Representative of the General Population?**			**Is the Assessment of the Outcome Accurate both at Baseline and at Follow-Up?**				**Is There Little Missing Data?**		
Martinez et al., 2017 [13]			−			+				−		
Burrello et al., 2011 [12]			−							−		
De Mas et al., 2001 [11]			−									

Yes: +; Cannot tell: Blank; No: −.

## Data Availability

Not applicable.
